# Comparison of Conformational Phase Behavior for Flexible and Semiflexible Polymers

**Published:** 2022-12-02

**Authors:** Dilimulati Aierken, Michael Bachmann

**Affiliations:** 1 Soft Matter Systems Research Group, Center for Simulational Physics, The University of Georgia, Athens, GA 30602, USA

**Keywords:** flexible polymers, semiflexible polymers, conformational phases, phase transitions, Monte Carlo simulations, microcanonical analysis

## Abstract

We employ the recently introduced generalized microcanonical inflection point method for the statistical analysis of phase transitions in flexible and semiflexible polymers and study the impact of the bending stiffness upon the character and order of transitions between random-coil, globules, and pseudocrystalline conformations. The high-accuracy estimates of the microcanonical entropy and its derivatives required for this study were obtained by extensive replica-exchange Monte Carlo simulations. We observe that the transition behavior into the compact phases changes qualitatively with increasing bending stiffness. Whereas the Θ collapse transition is less affected, the first-order liquid-solid transition characteristic for flexible polymers ceases to exist once bending effects dominate over attractive monomer-monomer interactions.

## I. INTRODUCTION

Statistical analysis methods have long been used for the identification, characterization, and classification of phase transitions in complex systems. Advanced computer simulation techniques, mostly based on Markov chain Monte Carlo algorithms, helped advance the field beyond the deadlocks mathematical approaches had run into. Nonetheless, the general idea remained the same. Phase transitions were generally only considered in the thermodynamic limit, which was historically introduced to make studies of macroscopic systems mathematically tractable. In computational statistical physics, finite-size scaling analysis provided the tool for extrapolating the results obtained in simulations of systems of finite size toward this hypothetical limit. These methods proved to be extremely successful, and for decades the scientific community did not see any reason to change paradigms and consider a more general perspective despite the fact that all systems in nature are finite and systems on nanoscales moved into the focus of substantial interest in microbiology and technology. Ignoring surface effects was also convenient.

Consequently, conventional canonical statistical analysis of phase transitions rests on the search for catastrophic behavior of thermodynamic state variables and response functions in thermodynamic parameter spaces. Hence, significant effort has been dedicated to the evaluation of critical exponents in continuous phase transitions and the grouping of systems in universality classes.

In recent years, however, the growing interest in systems on microscopic and mesoscopic scales, which do not satisfy the criteria for the thermodynamic limit, has shifted the perspective. Alternative approaches that enable studies of phase transitions in systems of any size have turned out to be promising. Modern interdisciplinary research of systems on nanometer scales cannot ignore the fact that the surface of the system is at least as important for the structural behavior of the system as the bulk effects. For example, the functionality of biomacro-molecules like proteins depends on the folding process into stable geometric conformations. These processes, which occur in a thermal environment, resemble transitions between disordered and ordered phases known from macroscopic systems. In fact, for very long polymers, these structure formation processes *are* phase transitions, even in the strict conventional sense. However, many types of heterogeneous polymers like proteins cannot be scaled up, but still show clear transition features. This makes the extension of the established theory of phase transitions a necessity.

However, canonical statistical methods create ambiguities, which render the unique characterization of phase transitions problematic. A prominent example is the peak in the specific heat curve of the one-dimensional Ising model. As it is also finite in the thermodynamic limit, one does not associate a phase transition with it. But how to interpret a peak in response quantities, if the thermodynamic limit for this system does not exist? In recent years, it has nonetheless become common to consider peaks or “shoulders” transition signals in curves of response quantities for finite systems. This appears to be an inconsistent approach and renders, in fact, the choice of the temperature as the basic thermodynamic state variable questionable.

Combining results of previous studies of the microcanonical Boltzmann entropy [1] and the principle of minimal sensitivity [2, 3], we recently developed a systematic approach to phase transitions in systems of any size by identifying least-sensitive inflection points in the entropy and its derivatives [4]. This analysis method even allows for the classification of phase transitions in analogy to Ehrenfest’s classification scheme in thermodynamics, which is based on derivatives of thermodynamic potentials [5].

In this paper, we extend previous Monte Carlo computer simulation studies of a coarse-grained model for flexible polymers [6] and analyze structural transitions in semiflexible polymers by means of the generalized microcanonical least-sensitive inflection point method. We thoroughly investigate the changes of phase behavior of a coarse-grained model for flexible and semiflexible polymers.

The paper is organized as follows. In Sec. II, we describe the flexible and semiflexible variants of the coarse-grained polymer model used in this study, as well as the numerical methods for the stochastic simulation of the model and for the statistical analysis of the data obtained in the simulations. The results of the canonical and microcanonical analysis are discussed and the phase behavior of the polymers is interpreted in Sec. III. This section also contains a detailed comparison of geometric properties of the lowest-energy conformations found for each system. Eventually, the paper is concluded by the summary in Sec. IV.

## II. MODELS AND METHODS

For this study we employed a generic coarse-grained model for polymers in implicit solvent that allows us to investigate and compare the phase behavior of flexible and semiflexible polymer chains by means of advanced Monte Carlo computer simulation methodologies. Model, simulation methods, and the microcanonical inflection-point technique we used for the statistical analysis of the conformational transitions are described in the following.

### A. Generic model for flexible and semiflexible polymers

The total energy of a polymer chain with *N* monomers is composed of contributions from bonded and non-bonded interactions between monomers. For the interactions between non-bonded monomers, we employ the standard 12–6 Lennard-Jones (LJ) potential:

(1) $$V_{\text{LJ}}(r)=4\epsilon_{\text{LJ}}\left[{\left(\frac{\sigma }{r}\right)}^{12}-{\left(\frac{\sigma }{r}\right)}^{6}\right].$$

Here, *r* is the monomer-monomer distance, *σ* = 2^−1/6^*r*_0_ is the van der Waals distance associated with the potential minimum at *r*_0_, and *∈*_LJ_ is the energy scale. For computational efficiency, we introduce a cutoff at *r*_*c*_ = 2.5*σ*. Shifting the potential by the constant *V*_shift_ = *V*_LJ_(*r*_*c*_) avoids a discontinuity at *r*_*c*_. Thus the potential energy of non-bonded monomers is given by

(2) $$V_{\text{NB}}(r)=\{\begin{array}{ll} V_{\text{LJ}}(r)-V_{\text{shift }}, & r<r_{c}\\ 0, & \text{ otherwise}. \end{array}$$

The elastic bond between two neighboring monomers is modeled by a potential which combines a Lennard-Jones potential and the finitely extensible nonlinear elastic (FENE) potential [7–9]:

(3) $$V_{\text{B}}(r)=-\frac{1}{2}K_{\text{f}}R^{2}\text{ln }\left[1-{\left(\frac{r-r_{0}}{R}\right)}^{2}\right]+V_{\text{LJ}}(r)-V_{\text{shift}},$$

where the FENE parameters are fixed to standard values *R* = (3/7)*r*_0_ and *K*_f_ = (98/5)*∈*_LJ_/*r*_0_^2^ [10]. The bond length *r* is restricted to fluctuations within the range [*r*_0_ − *R*, *r*_0_ + *R*]. With these parameters the minimum of *V*_B_ is located at *r*_0_.

For semiflexible polymers, an additional potential related to the bending stiffness is introduced. Any deviation from the reference angle *θ*_0_ between neighboring bonds is subject to an energy penalty of the form:

(4) $$V_{\text{bend}}(\theta )=\kappa \left[1-\text{cos}\left(\theta -\theta_{0}\right)\right].$$

If we represent a conformation of a polymer chain with *N* monomers by ***X*** = (***r***_1_, …, ***r***_*N*_), where ***r***_*i*_ is the position vector of the *i*th monomer, the total energy of the polymer is given by

(5) $$E(X)=\underset{i>j+1}{\sum }V_{\text{NB}}\left(r_{i,j}\right)+\underset{i}{\sum }V_{\text{B}}\left(r_{i,i+1}\right)+\underset{l}{\sum }V_{\text{bend}}\left(\theta_{l}\right),$$

where *r*_*i*, *j*_ = |***r***_*i*_−***r***_*j*_| is the distance between monomers *i* and *j*, and *θ*_*l*_ is the bond angle between two adjacent bonds. The parameter *κ* ≥ 0 controls the stiffness of the polymer chain. For *κ* = 0, the model describes flexible polymers.

In simulations and statistical analysis of the results, we set the basic scales to the following values: *k*_B_ = 1 (Boltzmann constant), *∈*_LJ_ = 1, and *r*_0_ = 1. For the reference bending angle, we chose *θ*_0_ = 0. The flexible chain with *N* = 55 monomers has already been studied extensively in the past and serves as the reference for the comparison with the semiflexible model. This chain length is sufficiently short to recognize finite-size effects, but long enough for the polymer to form a stable solid phase at low temperatures [11].

### B. Replica-Exchange Monte Carlo method and multiple-histogram reweighting

For the simulations of the polymer, we employed the replica-exchange (parallel tempering) Monte Carlo method [12–16]. Replicas of the systems were simulated at different temperatures *T*_*k*_ ∈ [0.1, 5.0] with *k* = 1, 2, …, *K*. The total number of temperature threads *K* ranged from 40 to 50 in individual simulations. Up to ten independent runs were performed for all *κ* values studied, which allowed for the estimation of numerical errors in all microcanonical quantities.

At each temperature *T*_*k*_, Metropolis sampling was performed, which is based on the acceptance probability:

(6) $$P\left(E_{k},T_{k};E_{k}^{\prime },T_{k}\right)=\text{min }\left\{e^{-\left(E_{k}^{\prime }-E_{k}\right)/k_{\text{B}}T_{k}},1\right\}.$$

Here *k*_B_*T*_*k*_ is the the thermal energy at the simulation temperature *T*_*k*_, $E_{k}^{\prime }$ is the energy of the proposed state, and *E*_*k*_ is the energy of the current conformation.

Local structural changes were achieved by displacement moves. In this Monte Carlo update, a monomer *i* is randomly picked and its position is modified by a random shift Δ***r***_*i*_ within a cubic box with edge lengths *r*_*d*_ surrounding the monomer. Before measurements can be performed, *r*_*d*_ is determined adaptively for each temperature thread to achieve a Metropolis acceptance rate of about 50%.

In order to explore the conformation space more efficiently, rotational pivot updates were also used. After 20 to 70 sweeps of displacement updates (depending on the temperature), we performed a sweep of pivot rotational updates. In a pivot update, a monomer *i* is randomly chosen as the pivot point and a rotation axis vector ***s***_*i*_. Then the section of the chain following the pivot monomer was rotated about ***s***_*i*_ by a randomly chosen angle taken from the interval [0, 2*π*].

Replicas were swapped between neighboring threads *k* and *k* + 1 after every 1500 sweeps with the exchange probability:

(7) $$P\left(E_{k},T_{k};E_{k+1},T_{k+1}\right)=\text{min }\left\{\text{exp }\left[\left(E_{k}-E_{k+1}\right)\left(\frac{1}{k_{\text{B}}T_{k}}-\frac{1}{k_{\text{B}}T_{k+1}}\right)\right],1\right\},$$

where *E*_*k*_ and *E*_*k*+1_ are the energies of the replicas before the swap at temperatures *T*_*k*_ and *T*_*k*+1_, respectively.

In order to estimate the density of states of the system, we use the multi-histogram reweighting method [17, 18]. For this purpose, we measured the canonical histograms in each thread. By utilizing the canonical distribution function *P*_can_(*E*; *T*_*k*_) ~ *g*(*E*)exp{−*E*/*k*_B_*T*_*k*_}, estimates for the density of states can be obtained by reweighting the energy histograms *h*(*E*; *T*_*k*_) measured in the different simulation threads *k*, $\bar{g}(E)=h\left(E;T_{k}\right)\text{ exp}\left\{E/k_{\text{B}}T_{k}\right\}$. Using a single histogram only covers a narrow range of energies effectively. Combining the histograms obtained at different temperatures by employing the multi-histogram reweighting method yields an estimator for the density of states that covers the entire energy range [17, 18]:

(8) $$\hat{g}(E)=\frac{\sum_{k=1}^{K}h\left(E;T_{k}\right)}{\sum_{k=1}^{K}M_{k}Z_{k}^{-1}e^{-E/k_{\text{B}}T_{k}}}.$$

Here *M*_*k*_ is the total number of sweeps in the thread *k* and *Z*_*k*_ is an estimator for the partition function:

(9) $$Z_{k}=\underset{E}{\sum }\hat{g}(E)e^{-E/k_{\text{B}}T_{k}}.$$

Equations (8) and (9) are solved iteratively until reasonable convergence has been achieved.

### C. Generalized microcanonical inflection-point analysis method

Historically, phase transitions have been identified and classified by means of discontinuities or divergences in thermodynamic state variables or response functions. However, systems of finite size do not exhibit such obvious signals, as these only occur in the thermodynamic limit. In this study, we use the recently introduced generalized microcanonical inflection-point analysis method [4] for the systematic identification and classification of transitions in systems of any size. This method, which combines microcanonical thermodynamics [1] and the principle of minimal sensitivity [2, 3], has already led to novel insights into the nature of phase transitions. Even the Ising model, which has been excessively studied in almost a century, possesses a more complex phase structure than previously known, as our most recent analysis showed [19].

Adopting Boltzmann’s formula, the microcanonical entropy can be written as

(10) $$S(E)=k_{\text{B}}\text{ ln }g(E),$$

where *g*(*E*) is the density of states with energy *E*. When not experiencing phase transitions, the curves of entropy *S*(*E*) and its derivatives exhibit well-defined concave or convex monotony [4]. From canonical statistical analysis of first- and second-order transitions, it is known that entropy and/or internal energy rapidly change, if the temperature is varied near the transition point. This behavior corresponds to least-sensitive dependencies of microcanonical quantities in the space of system energies. Thus, a phase transition causes a least-sensitive inflection point in the entropy or its higher derivatives and impacts their monotonic behavior. By systematically analyzing these alterations, different types of transitions can be identified and classified.

In this scheme, a first-order transition is signaled by a least-sensitive inflection point with energy *E*_tr_ in *S*(*E*). Therefore, the first derivative, which is the inverse microcanonical temperature *β*(*E*), possesses a positive valued minimum at *E*_tr_,

(11) $$\beta \left(E_{\text{tr}}\right)={\frac{\text{d}S(E)}{\text{d}E}|}_{E=E_{\text{tr}}}>0.$$

Consequently, if there is a least-sensitive inflection point in *β*(*E*), the phase transition is classified as a second-order transition. The derivative of *β*(*E*) has a negative-valued peak at the transition energy *E*_tr_,

(12) $$\gamma \left(E_{\text{tr}}\right)={\frac{{\text{d}}^{2}S(E)}{\text{d}E^{2}}|}_{E=E_{\text{tr}}}<0.$$

More specifically, we call this an *independent* second-order transition. This implies that there is another transition type: *dependent* transitions. As the name suggests, dependent transitions are associated with an independent transition of lower-order rank. It is important to note that not all independent transitions have a dependent companion. However, if it exists - and it can only exist at a higher transition energy than its independent partner — it servers as a precursor of the independent transition in the disordered phase. For example, if this inflection point is located within the convex region of the entropy, which is associated with the first-order transition, the transition is a dependent second-order transition. Consequently, there is a positive-valued minimum in the derivative of *β*(*E*) at the transition energy *E*_tr_,

(13) $$\gamma \left(E_{\text{tr}}\right)={\frac{{\text{d}}^{2}S(E)}{\text{d}E^{2}}|}_{E=E_{\text{tr}}}>0.$$

Generally, for an independent transition of odd order (2*k* − 1) (*k* is a positive integer), the (2*k* − 1)th derivative of *S*(*E*) possesses a positive-valued minimum,

(14) $${\frac{{\text{d}}^{\left(2k-1\right)}S(E)}{\text{d}E^{\left(2k-1\right)}}|}_{E=E_{\text{tr}}}>0$$

and an independent transition of even order 2*k* is characterized by a negative-valued peak in 2*k*th derivative,

(15) $${\frac{{\text{d}}^{2k}S(E)}{\text{d}E^{2k}}|}_{E=E_{\text{tr}}}<0.$$

A dependent transition of even order is identified by a positive-valued minimum in the 2*k*th derivative,

(16) $${\frac{{\text{d}}^{2k}S(E)}{\text{d}E^{2k}}|}_{E=E_{\text{tr}}^{\text{dep}}}>0,$$

while for odd order 2*k* + 1:

(17) $${\frac{{\text{d}}^{\left(2k+1\right)}S(E)}{\text{d}E^{\left(2k+1\right)}}|}_{E=E_{\text{tr}}^{\text{dep}}}<0.$$

Dependent transitions are less common than independent transitions. In this polymer study we did not identify any dependent transitions.

We used the Bézier method [20–22] to smooth curves and calculate derivatives.

## III. RESULTS

### A. Canonical analysis

In the past, the common approach to the characterization of phase transitions has been canonical statistical analysis. For infinitely large systems, one or more derivatives of an appropriate thermodynamic potential such as the free enthalpy or the free energy would exhibit nonanalytic behavior, if one of the potential’s natural variables was altered at the transition point. Because it is easy to control in experiment, the analysis has typically been done in the space of the canonical (or heat bath) temperature *T*_can_. Discontinuities in the canonical entropy and specific heat help identify and characterize discontinuous and continuous phase transitions, respectively, in the thermodynamic limit. Later, this approach was simply extended to systems of finite size that do not allow for the hypothetical extrapolation toward the thermodynamic limit (for example, heterogeneous polymers such as proteins).

However, transition features in finite systems cannot be linked to nonanalyticities. Hence, *peaks* and *shoulders* in response quantities like heat capacity or order parameter fluctuations have usually been considered signals of pseudotransitions in finite systems. The problem with this approach is that extremal points in fluctuating quantities are not safe indicators of phase transitions. The probably most prominent example is the specific-heat curve for the one-dimensional Ising model, which exhibits a pronounced maximum at a certain temperature, but there is no obvious feature that links this extremal thermal activity to a phase transition. In the thermodynamic limit, this peak does not develop into a divergence.

Interestingly, if one plots the heat capacity for the 1D Ising system as a function of the *inverse temperature β* = 1/*T*, though, the peak vanishes and there is no pseudotransition signal. Note that in the microcanonical analysis the inverse temperature *β*(*E*) = *dS*(*E*)/*dE* is the more obvious thermodynamic variable than the temperature. Consequently, microcanonical inflection-point analysis does not show any transition signal for the 1D Ising system in equilibrium [19].

In fact, historically the (canonical) temperature was introduced when the need arose to assign a reliable scale to the thermoscope to quantify the environmental heat content, which ultimately lead Galilei to the introduction of the thermometer at the end of the 16th century. It is ironic that the nowadays most commonly used centigrade scale, the Celsius scale, was introduced in 1742 by Celsius in *inverted* form, i.e., Celsius chose 0° as the boiling point of water under norm conditions and 100° as the reference point for the melting of ice. At about the same time, French scientist Christin independently used a centigrade scale that essentially inverted the original scale by Celsius, which is what we nowadays refer to as the *Celsius Scale* [23].

This brief historic recollection shows that the temperature and its scale were introduced in an era, when it was still believed to be a quantitative measure for heat, and the scale was ultimately linked to the instrument and substances used for its measurement. Thus, there is no physical reason that favors the historic quantity temperature over the inverse temperature, which is the more natural variable in the microcanonical analysis. To distinguish the microcanonical temperature from the heat bath temperature, we denote the latter by *T*_can_ in the following canonical analysis of structural transitions.

**FIG. 1.**
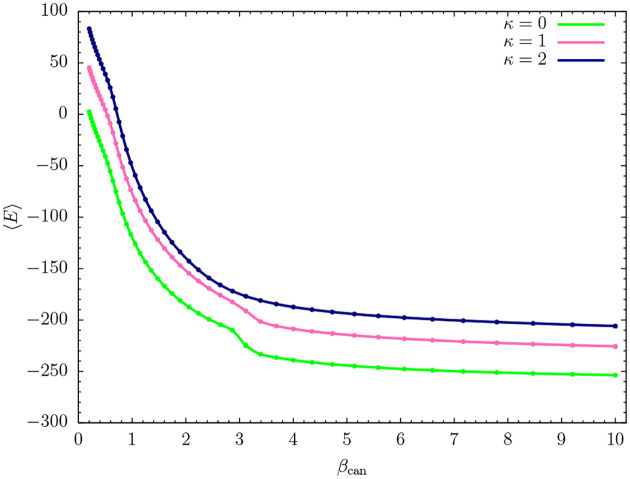
Canonical mean energy 〈*E*〉 as a function of *β*_can_ for flexible (*κ* = 0) and variants of semiflexible polymers with different bending stiffness (*κ* = 1, 2). Error bars are shown but smaller than the symbol sizes.

Consequently, we plot the canonical mean energy 〈*E*〉 as a function of *β*_can_ = 1/*T*_can_ in Fig. 1 for the three models studied: *κ* = 0 (flexible chain) and *κ* = 1, 2 (semiflexible variants). All curves show a sharp drop at *β*_can_ ≈ 0.7. Around *β*_can_ ≈ 3.2, the flexible chain (*κ* = 0) experiences another significant drop in energy, which is less pronounced for the semiflexible polymer with bending stiffness *κ* = 1. No obvious signal is visible on this level for the stiffer chain with *κ* = 2.

**FIG. 2.**
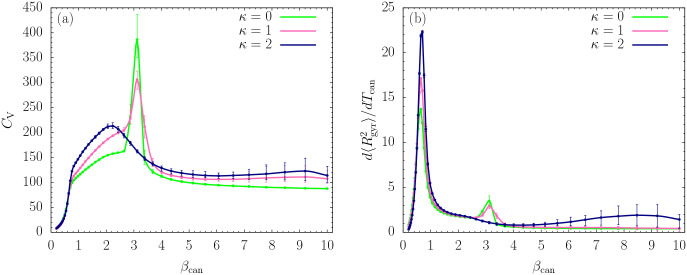
Thermal fluctuations of (a) energy (heat capacity *C*_*V*_ = *d*〈*E*〉/*dT*_can_) and (b) square radius of gyration $\left(d\langle R_{\text{gyr}}^{2}\rangle /dT_{\text{can}}\right)$, plotted as functions of *β*_can_.

The plots of fluctuating quantities, such as the heat capacity *C*_*V*_ = *d*〈*E*〉/*dT*_can_ and the fluctuations of the mean square radius of gyration, $d\langle R_{\text{gyr}}^{2}\rangle /dT_{\text{can}}$, allow for a more detailed analysis. Both quantities are shown as functions of *β*_can_ in Figs. 2(a) and 2(b), respectively. Whereas we certainly see changes in the curvature of the *C*_*V*_ curves at *β*_can_ ≈ 0.7, only the plots of the structural fluctuations show sharp peaks. These indicate the well-known Θ collapse transition between extended, random-coil structures and compact globular conformations. The transition signal is more pronounced for the stiffer chains. Since this transition is more entropy than energy driven, it is not surprising that it shows up more prominently in the structural rather than the energetic fluctuations. This is different for the freezing transition at about *β*_can_ ≈ 3.2, which is strongest for the flexible chain (*κ* = 0). Large error bars at very small temperatures (*β*_can_ ≈ 8.5) prevent a conclusion about another separate transition for the semiflexible polymer with *κ* = 2, which, if it exists, marks the transition into the global energy minimum basin.

### B. Microcanonical analysis of phase transitions

**FIG. 3.**
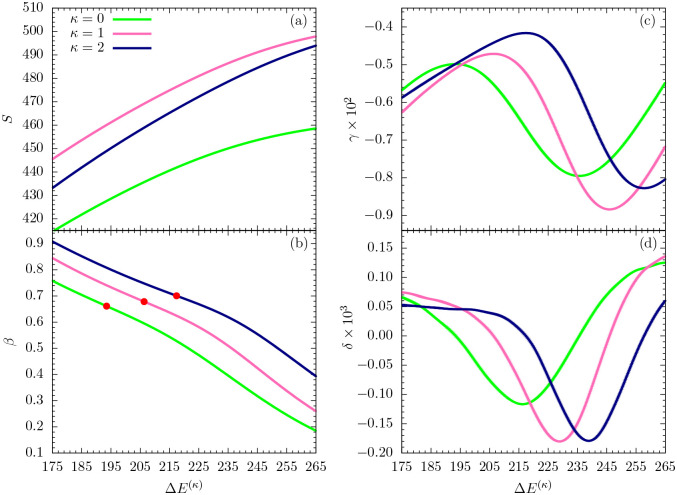
(a) Microcanonical entropy *S* and its derivatives (b) *β* = *dS*/*dE*, (c) *γ* = *dβ*/*dE*, (d) *δ* = *dγ*/*dE* for the different models with *κ* = 0, 1, 2 plotted as functions of the energy difference from the κ-dependent global energy minimum estimate. In this figure, we focus on the high-energy (or high-temperature) regime. Least-sensitive inflection points are marked by a dot.

**FIG. 4.**
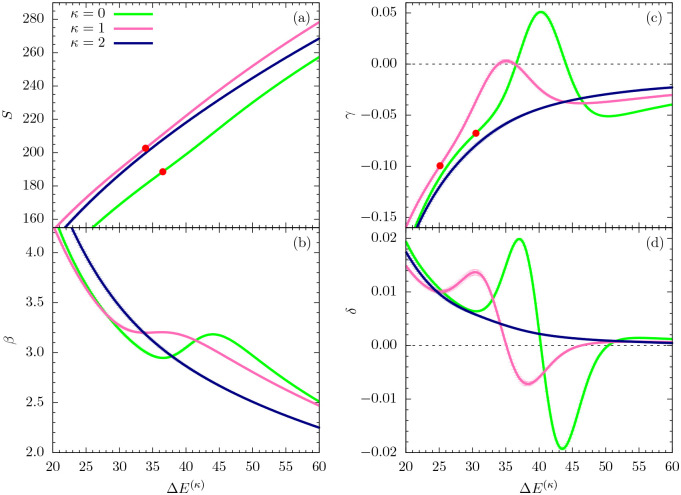
Same quantities as in Fig. 3, but plotted for an intermediate energy region.

**FIG. 5.**
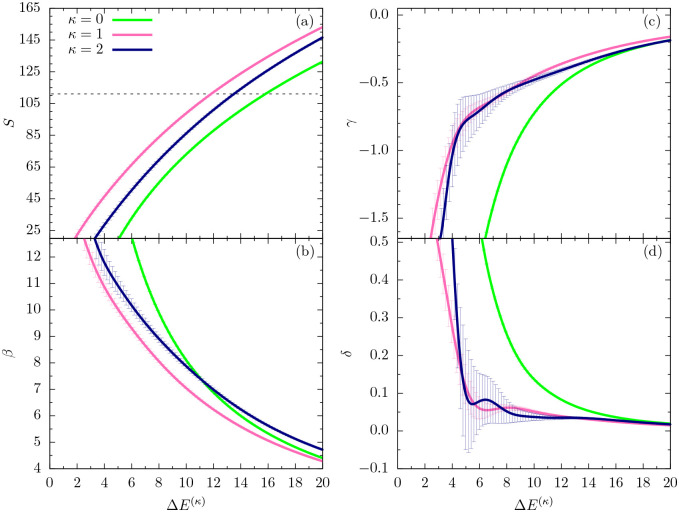
Plots of the same microcanonical quantities as in Figs. 3 and 4 in the lowest-energy regions of the three models.

In this section, we perform a full-scale microcanonical inflection-point analysis of the three models that aims at the identification of all structural transitions in these systems up to third order. Figures 3, 4, and 5 show the microcanonical entropy and its derivatives up to third order as functions of the reduced energy $\Delta E^{\left(\kappa \right)}=E-E_{\text{min}}^{\left(\kappa \right)}$, i.e., we subtracted from the energy the respective putative global energy minima $E_{\text{min}}^{\left(\kappa \right)}$ obtained for each system in the parallel tempering simulations and verified by simulated annealing. This shift allows for an easier comparison of the results.

As in the canonical analysis, we first discuss the low-*β* (or high-temperature) regime in the relevant energy space. The entropies plotted in Fig. 3(a) for all cases do not possess least-sensitive inflection points and thus there is no first-order transition, as expected. However, least-sensitive inflection points in the first derivative (*β*) signal second-order phase transitions in all three systems, which reflect the mostly entropic Θ collapse from random-coil to globular polymer structures. The corresponding peak locations in the second entropy derivative (*γ*), shown in Fig. 3(c), allow for a unique determination of the transition points in energy space and thus also in *β*, thereby rendering the ambiguous canonical analysis of response functions in the previous section obsolete. Since there are no least-sensitive inflection points in the *γ* plots, none of the systems undergoes a third-order transition in this energy region.

Figure 4 shows the same microcanonical quantities as plotted in Fig. 3, but for an intermediate energy range that covers the inverse temperatures in the interval *β* = (2,4). As expected for flexible polymers, the entropy curve for *κ* = 0 does exhibit a least-sensitive inflection point, which corresponds to the minimum in the backbending region found in the *β* plot [Fig. 4(b)] at about Δ*E*^(*κ*)^ = 36.5. The inverse temperature associated with it is approximately *β* = 2.95, which confirms earlier results for flexible polymers [6]. The polymer undergoes a freezing transition from the liquid-globular states into the solid phase, which for this model is dominated by icosahedral structures. Remarkably, for the semiflexible polymers with *κ* = 1, 2, the situation changes. For *κ* = 1, there is still a weak inflection point in the entropy, but the *β* curve has already almost plateaued at about the same energy value, where we found the first-order transition for the flexible polymer. Consequently, the peak value of the next derivative (*γ*) is virtually zero at this energy [Fig. 4(c)]. Therefore, the freezing transition seems to turn from first order for the flexible polymer (*κ* = 0) to second order for the semiflexible case with *κ* = 1. However, even more surprisingly, the transition behavior changes again for the stiffer chain with *κ* = 2: In fact, the transition signal has completely vanished. The subsequent structural analysis of ground-state properties will lend deeper insight into the reasons for these changes.

The second derivative *γ* in Fig. 4(c) shows signs of independent third-order transitions for *κ* = 0 and *κ* = 1 in the ordered phase, but not for *κ* = 2. These results suggest that freezing into a unique and characteristic global energy minimum state is not possible if bending effects overwhelm non-bonded interactions that are commonly responsible for tertiary structure formation in polymeric systems. Local bending effects turn into restraints that inhibit or at least suppress the formation of symmetries. Previous studies have shown that bending restraints play an important role in the formation of stable conformations in tertiary assemblies of helical segments [24].

Eventually, the sequence of figures shown in Fig. 5 covers the lowest-energy regime for all models compared in this study. In the flexible case, no additional transitions are expected and, correspondingly, we do not see signals in any of the derivatives up to third order that would indicate a transition. The situation is potentially different for the systems with bending restraint. Whereas we do not find indications of transition signals in the entropy and inverse temperature curves for *κ* = 1 and *κ* = 2 in Figs. 5(a) and 5(b), respectively, the *γ* results shown in Fig. 5(c) might tell a different story. The error bars are too large for an ultimate conclusion, but it looks like a third-order transition develops close to the ground state for the semiflexible polymer models.

The quantitative results of the microcanonical inflection-point analysis obtained for the polymer systems studied here are listed in Table I.

### C. Structural analysis of lowest-energy conformations

The lowest energies found in the simulations, which we consider best estimates of the ground state energies, are $E_{\text{min}}^{\left(0\right)}=-261.7$ for the flexible polymer (*k* = 0), $E_{\text{min}}^{\left(1\right)}=-230.9$ for the semiflexible chain with *κ* = 1, and $E_{\text{min}}^{\left(2\right)}=-212.4$ if *κ* = 2. All values are given in units of the energy scale of the Lennard-Jones potential, *∈*_LJ_. Since the bonded interactions (bond vibrations) are negligible near the ground state, the differences in the ground-state energy estimates for the different models must be attributed to the penalty paid for chain bending. The ground-state conformation for the flexible polymer that does not experience these restraints is formed by optimizing the non-bonded interaction and results, for this model, in an ideally icosahedral structure. Turning on the bending restraint and setting *κ* = 1, the competition between nonlocal attraction and local repulsion increases the tension in the icosahedral structure, although it still stays in shape. For *κ* = 2, however, it cannot maintain the optimal icosahedral arrangement of monomers and rather forms an entangled structure of longer, less bent, segments. As we have seen in the thermodynamic analysis of the transitions, the phase behavior changes significantly with increasing bending stiffness.

**FIG. 6.**
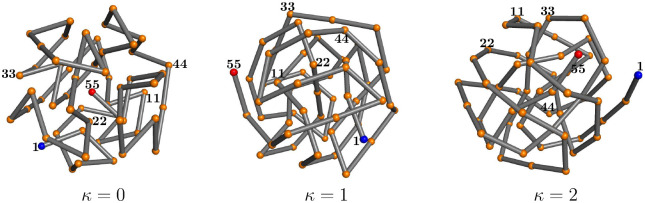
Representatives of lowest-energy conformations for *κ* = 0, 1, 2. Labels help to follow the chain from the first monomer (1, blue) to the last (55, red).

Representative lowest-energy conformations are shown in Fig. 6. For a more quantitative analysis let us take a look at the pair distribution functions and the contact maps. We introduce the pair distribution function as

(18) $$P(r)=\underset{i<j}{\sum }\Delta \left(r-r_{i,j}\right),$$

where

(19) $$[h]\Delta \left(r-r_{i,j}\right)=\{\begin{array}{ll} 1, & \left|r-r_{i,j}\right|<r_{t},\\ 0, & \text{ otherwise}. \end{array}$$

**FIG. 7.**
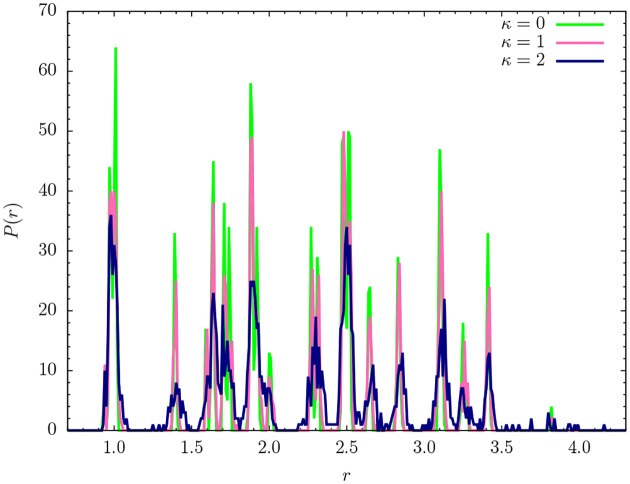
Pair distribution functions for the lowest-energy states shown in Fig. 6 for *κ* = 0, 1, 2.

As a robust threshold for the necessary binning of the *r* space, we chose *r*_*t*_ = 0.01. The histograms for the lowest-energy conformations shown in Fig 6 are plotted in Fig. 7. The perfect icosahedral structure is only found for *κ* = 0, whereas its decay is already visible for the weaker semiflexible polymer (*κ* = 1). The maximum number of nearest-neighbor contacts (*r*_*i*, *j*_ ≈ 1) found for *κ* = 0 is not reached in the semiflexible cases. The broadening of the peaks and additional spikes not present for *κ* = 0, 1 are clear indicators that the ground-state structure for the semiflexible polymer with *κ* = 2 is not icosahedral. Bending restraints prevent the formation of perfect symmetries and thus a characterization of the *κ* = 2 ground-state conformation as a distinct crystalline or quasicrystalline structure is not possible. It rather resembles tertiary folds of protein conformations, where effective bending restraints and the local stable secondary segments purposefully prevent symmetric arrangements of monomers. This enables different heteropolymers of similar size to form distinct and functional individual conformations.

**FIG. 8.**
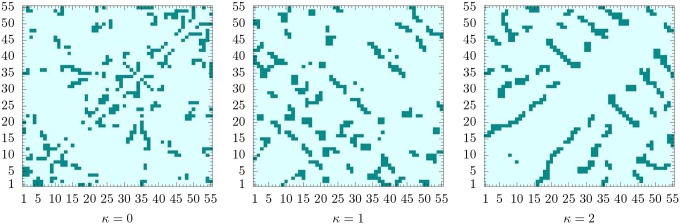
Contact maps for the lowest-energy states shown in Fig. 6 for *κ* = 0, 1, 2

This is obvious from the plots of the contact maps for the lowest-energy states shown in Fig. 8. In the grid spanned by the monomer labels, we mark pairs of monomers with distances *r*_*i*, *j*_ < 1.2. Bonded monomer pairs are not included. For the flexible polymer, the contact map does not exhibit particularly remarkable structural features. Since there are no bond angle restraints, each monomer tries to maximize its number of nearest neighbors for the energetic benefit. Icosahedral conformations with one of the end monomers in the center are optimal. For the semiflexible polymer with *κ* = 1, we already see that changes occur. Monomers do not only try to maximize the number of contacts, but three monomers connected by two adjacent bonds now have to cooperate to minimize bending. Close to the diagonal, we find that short anti-diagonal streaks form. These are clear indicators of turns with two linear strands in contact with each other (hairpins). In protein folds, these would be referred to as building blocks for *β*-sheets. For stronger bending rigidity (*κ* = 2), there are fewer, but longer such segments. We also observe the formation of streaks parallel to the diagonal, which can be associated with helical alignments. Both types of these secondary structure elements can be seen in the geometric representation in Fig. 6. The hairpin-like sections are located in the interior and the helical segments wrap around it. Of course, under these conditions, energetically favored structures are not icosahedral. In fact, no global symmetries can be identified in this tertiary fold. Although it is tempting to think of these structures in analogy to protein folds, the lack of dihedral (torsion) constraints, which separates secondary structure elements from each other in the tertiary fold, prevents a more direct comparison.

## IV. SUMMARY

The statistical analysis of structural transitions by the recently developed generalized microcanonical inflection-point method [4] had already yielded promising results in studies of flexible polymers [6]. For this study, we extended the coarse-grained model by incorporating bending restraints of different strengths to investigate the impact of bending stiffness on the microcanonical entropy and its derivatives, which are used as indicator functions for the systematic identification and classification of phase transitions in systems of any size. In this coarse-grained model for semiflexible polymers, the bending restraint is controlled by the bending stiffness parameter *κ*.

Despite its simplicity, the model required a careful numerical treatment. For this purpose, we performed extensive parallelized replica-exchange computer simulations and verified the structural behavior in the lowest-energy basin by means of stochastic methods optimized for this purpose such as simulated annealing. Microcanonical thermodynamic properties were obtained from the density of states, which is the main output of the multiple-histogram reweighting method applied to the raw simulation data.

Based on the microcanonical results obtained in the simulations, we compared the phase behavior of three model variants: purely flexible polymers (*κ* = 0) and semiflexible polymers with *κ* = 1 (identical energy scales of attractive non-bonded monomer-monomer interactions and repulsive bending strength) and *κ* = 2 (bending restraints dominate over monomer-monomer attraction). Our simulations reproduced previous results for the flexible reference system very well, creating sufficient confidence for the subsequent studies of the semiflexible polymers. Because of the additional restraints, it was significantly more challenging to achieve in simulations of semiflexible polymers the data quality necessary to enable an accurate microcanonical analysis.

For the flexible polymer, we identified the known structural transitions, i.e., the characteristic second-order transition associated with the Θ collapse from extended random coils to liquid globules and the first-order liquid-solid transition, which is accompanied by an independent third-order transition. The lowest-energy conformation is icosahedral, as expected. The behavior does not change qualitatively for the weaker semiflexible case with *κ* = 1, where the effectively repulsive bending effects are competitive to attractive monomer-monomer interaction. However, the larger number of possible attractive monomer-monomer contacts represents an entropic advantage and thus the icosahedral solid phase is still maintained. This is not the case anymore if the chain is further stiffened by choosing *κ* = 2. Whereas the Θ collapse remains widely unaffected, there is no obvious transition into the solid phase anymore. Signals at very low energies at the third-order transition level are too noisy to providea clear picture. However, it is obvious that the lowest-energy conformation found is a compromise of satisfying both compactness and bending restraint. Longer, only slightly bent segments form and assemble in a well-organized, coil-like structure. It is an intriguing future task to extend our microcanonical study to stronger bending restraints and to construct the complete hyperphase diagram in the spaces of bending stiffness and inverse temperature and to apply it to other modern applications in polymer science like hyperbranched polymers [25].

## Figures and Tables

**TABLE I.** Transitions found from microcanonical analysis for different flexible (*κ* = 0) and semiflexible (*κ* = 1, 2) polymers. Transition energy *E*_tr_, distance from putative ground-state energy Δ*E*^(*κ*)^, inverse microcanonical transition temperature *β*_tr_, and the order of the transition (classification) are listed.

Bending stiffness	*E*_tr_	Δ*E*^(*κ*)^	*β*_tr_	Classification
*κ* = 0	−231.2	30.5	3.20	3
	−225.2	36.5	2.95	1
	−68.2	193.5	0.66	2
*κ* = 1	−205.8	25.1	3.63	3
	−197.0	33.9	3.20	1
	−24.6	206.3	0.68	2
*κ* = 2	5.0	207.4	0.70	2
